# Health related quality of life of gynaecologic cancer patients attending at Tikur Anbesa Specialized Hospital (TASH), Addis Ababa, Ethiopia

**DOI:** 10.1186/s12905-017-0507-7

**Published:** 2018-01-05

**Authors:** Birhanu Abera Ayana, Shiferaw Negash, Lukman Yusuf, Wendemagegnhu Tigeneh, Demewoz Haile

**Affiliations:** 1 Department of Gynecology & Obstetrics, College of Medicine and Health Sciences, University of Gondar, Gondar, Ethiopia; 2 Department of Gynecology &Obstetric, School of Medicine, College of Health sciences, Addis Ababa University, Addis Ababa, Ethiopia; 3 Department of Oncology, School of Medicine, College of Health sciences, Addis Ababa University, Addis Ababa, Ethiopia; 4 Department of Reproductive Health and Health Service Management, School of Public Health, College of Health sciences, Addis Ababa University, Addis Ababa, Ethiopia

**Keywords:** Gynaecologic cancer, Quality of life, EORTC QLQ-C30, Ethiopia

## Abstract

**Background:**

Being diagnosed with gynaecologic malignancy certainly will have different sequelae which can hamper quality of life (QOL).This study aimed to assess health related quality of life (HRQOL) among gynaecologic cancer patients attending at Tikur Anbesa Specialized Hospital (TASH), Addis Ababa, Ethiopia.

**Methods:**

This study employed facility-based cross-sectional study design on 153 gynaecological cancer patients attending TASH using the Amharic version of the European Organization for Research and Treatment of Cancer Quality of Life Core Questionnaire (EORTC QLQ-C30). We used descriptive statistics, independent t test and one way analysis of variance (ANOVA) in statistical analysis.

**Results:**

The mean Global Health Status (GHS) was 40.95(SD ± 24.35) and of the functional scores, social function was most affected (42.26, SD ± 32.08), whereas cognitive function is the least affected domain (mean = 88.21, SD ± 18.49). The highest score on the symptom scores was found to be financial difficulties (mean = 64.76, SD ± 32.43) followed by pain (mean = 55.12, SD ± 29.64) and fatigue (mean = 53.97, SD ± 28.54); the lowest score on the contrary was scored for diarrhea (mean = 1.19, SD ± 7.38). As stage increases there was a statistically significant reduction in GHS (*p* = 0.005) and in all functional score domains except the physical and emotional function. Advancement in stage of the disease has also affected significantly the symptom score domains except financial difficulties, nausea /vomiting and diarrhea. Patients who never went to school have scored a statistically significant lower score in GHS, physical function, role function and social function (*p* < 0.05).

**Conclusion:**

GHS, social function, financial difficulties, pain and fatigue were the most affected domains; however, cognitive function and diarrhea were less affected components of HRQOL of gynaecologic cancer patients. Place of residence, educational status, marital status, payment type, cancer type and stage of the disease were associated with different quality of life scores.

## Background

Quality of life (QOL) is a new dimension of care which has received greater attention in the last three decades. QOL is a multidimensional concept which is defined as a person’s view of life, and with her satisfaction and pleasure with life [1]. QOL is concerned with social, emotional and physical well-being of patients following treatment, mirroring the World Health Organization’s (WHO’S) definition of health [2].

Being diagnosed with gynaecologic malignancy certainly will have different sequelae which can hamper QOL. The special thing for gynaecological cancers is its effect on reproductive performance, sexuality and body image [3]. Understanding these impacts is vital to improve approaches to care, modify therapies and provide supportive care for the duration of the illness and enhance QOL.

Assessment of QOL among gynaecologic cancer patients provides supplementary information for physicians for selecting antineoplastic and supportive-care therapy. Managing QOL in gynaecologic cancer patients requires careful consideration of a variety of issues, many of which revolve around the surgical procedures employed and major side effects induced by the therapeutic agents used, as well as disease-associated factors that can negatively affect QOL [4].

Taking into consideration the chronic often incurable nature of the disease and the high risk of recurrence, cancer has a significant effect on QOL. The diagnosis by itself can cause different psychological problems like anxiety, fear, anger, sadness, and depression which compromise QOL [5]. Though gynaecologic cancer constitutes a large proportion of female cancers in Ethiopia, HRQOL of gynaecologic cancer patients is not assessed yet. Therefore this study aimed to investigate HRQOL of gynaecologic cancer patients using the Amharic version of EORTC QLQ-C30 attending at TASH, Addis Ababa, Ethiopia.

## Methods

### Study setting

This study was conducted at the oncology center of Tikur Anbesa Specialized Hospital, Addis Ababa, Ethiopia. The hospital is a teaching hospital of Addis Ababa University and a major referral center from all corners of the country especially for cancer patients. The hospital has a variety of specialty and sub-specialty training in varies fields of study including gynaecology/obstetrics, surgery and oncology. The hospital owned the only oncology center in the nation providing radiation therapy during the study period. The oncology center has about 500 adult and pediatric cases with hematologic malignancies every year. The most common adult cancers were cervical, breast, sarcomas, head and neck, and colorectal cancers.

### Study design and period

This study employed a facility based cross-sectional study on gynaecologic cancer patients attending TASH between January 1st to June 30^th^2014. The study team approached a total of 153 gynaecologic cancer patients. However 13 patients were excluded from the study because they refused to provide information on the variables of interest of this study after they gave their consent participate.

### Quality of life assessment tool

The quality of life was assessed by Amharic version of EORTC QOL-C30 [6]. The study group evaluated the reliability and validity of the questionnaire and found the reliability and validity of the tool was in acceptable range despite its few limitations [7].

### Study subjects

We only included adult gynaecologic cancer patients’ age ≥ 18 years and who were taking oncologic treatment for the first time. Those adult gynaecologic cancer patients who had received oncological treatment previously were excluded from study because the treatment by itself can compromise QOL significantly. We also excluded those patients with psychiatric disorders, communication disorders, severe other medical illnesses or diagnosed with coexisting malignancies and HIV positive patients because all this health problems compromise the quality of life which could confound the impact of the cancer on quality of life.

### Statistical analysis

Before the data were entered to SPSS version 20 for windows, it was checked for completeness, inconsistencies and cleaned by the study team. The team also did data cleaning on the entered data and double-check its consistency with the paper questionnaire. The raw scores were transformed to scores ranging from 0 to 100. Linear transformation to 0-100 to obtain the score S, have been done according to the formula given by EORTC [6].

Descriptive statistics (frequencies, mean, and standard deviation) were used to summarize the data. Bivariate analyses (independent t test and one way ANOVA) were used to determine the association between the QOL and patient socio-demographic and clinical characteristics. A *p* value of <0.05 was used to declare a statistically significant association.

## Results

### Socio-demographic and clinical characteristics of patients

In this study, the study team approached a total of 153 study subjects; however only 140 gynaecologic cancer patients who had completed data included in the analysis. The socio-demographic characteristics of the gynaecologic cancer patients included in this study were depicted in our published article of the validity section of this study [7].

Cervical cancer represents the most frequent cancer type in the study, diagnosed in 77.2% of patients and it was followed by ovarian and endometrial cancer (15% and 5% respectively). The majority of patients 55(47%) were either at stage 3 or 4, and followed by stage two 49(41.9%) (Table 1).

**Table 1:** Clinical characteristics of gynaecological cancer patients attending treatment at TASH, Addis Ababa, Ethiopia, 2014

Clinical characteristics		Frequency	Percent
Cancer type	Cervical	108	77.2
	Ovarian	21	15.0
	Endometrial	7	5.0
	Others(vaginal, vulvar)	4	2.8
Stage of the diseases (*n* = 117)	1	12	10.3
	2	49	41.9
	3 and 4	55	47.0
Treatment type	Radiation	84	60.0
	Operation	56	40.0
Payment type	Paying	103	73.6
	Free	37	26.4
Care givers relation	Children	96	68.6
	Spouse	25	17.9
	Sibling	10	7.1
	Parents	9	6.4

### Quality of life scores

The mean Global Health Status (global quality of life) score was 40.95(SD **±** 24.35) and of the functional scales, social function 42.26 (SD ± 32.08), was the most affected domain followed by role function 50.12 (SD ± 35.11) whereas cognitive function is least affected (mean = 88.21, SD **±** 18.49). The highest score on the symptom scale was for financial difficulties (mean = 64.76, SD **±** 32.43) followed by pain (mean = 55.12, SD **±** 29.64) and fatigue (mean = 53.97, SD **±** 28.54) while the lowest score was for diarrhea (mean = 1.19, SD **±**7.38) **(**Table 2).

**Table 2:** Mean and standard deviation of EORTC- QLQ C-30 components for gynaecological cancer patients at TASH, Addis Ababa, Ethiopia, 2014

EORTC- QLQ C-30 components	Items	Mean	Std. Deviation
GHS	29,30	40.95	24.35
Functional scale			
Physical function	1 – 5	65.24	22.59
Role function	6,7	50.12	35.11
Emotional function	21 – 24	55.48	30.32
Cognitive function	20, 25	88.21	18.49
Social function	26, 27	42.26	32.08
Symptom scale			
Fatigue	10, 12, 18	53.97	28.54
Nausea and Vomiting	14, 15	8.21	17.92
Pain	9, 19	55.12	29.65
Dyspnea	8	6.67	17.97
Insomnia	11	36.19	33.57
Appetite loss	13	44.05	36.71
Constipation	16	44.29	39.06
Diarrhea	17	1.19	7.38
Financial difficulties	28	64.76	32.43

### Association of quality of life scores with socio-demographic and clinical characteristics

As stage increases there is a statistically significant reduction in GHS (*p* = 0.005) and in all functional scale domains [physical function (*p* = 0.002), role function (*p* = 0.032) and cognitive function (*P* < 0.001)] except social function (*p* = 0.065) and emotional function (*p* = 0.149) (Table 3). Advancement in stage of the disease has also affected significantly the symptom scale domains such as fatigue (*p* < 0.001), pain (*p* < 0.001), insomnia (*p* = 0.002), and constipation (*p* < 0.001) and appetite loss (*p* = 0.013). But it was not significantly associated with financial difficulties (*p* = 0.32), nausea/vomiting (*p* = 0.35) and diarrhea (*p* = 0.87) (Table 4).

**Table 3:** EORTC QLQ-C30 functional scores by socio-demographic and clinical characteristics of gynaecological cancer patients at TASH Addis Ababa, Ethiopia, (*n* = 140)

Variables		GHS	Physical function	Emotional function	Role function	Social function	Cognitive function
Stage of the diseases	Stage 1	54.86(±26.46	82.78(18.52	62.50(±30.26)	66.67(±34.82	48.61(±35.15	93.06(±16.60)
	Stage 2	44.90(±20.04)	67.35(±19.59)	59.86(±28.29)	54.42(±34.66)	45.92(±33.34)	90.82(±13.64)
	Stage 3 and 4	34.39(±24.32)	56.96(±24.44)	48.79(±31.07)	38.79(±34.99)	33.03(±29.48)	84.24(±21.85)
	*P*-value	0.005*	0.002*	0.149	0.032*	0.065	<0.001*
Address	Addis Ababa	45.09(26.47)	67.35(22.21)	58.76(±29.7)	53.42(±32.03)	51.71(±34.5)	88.89(15.45)
	Out of Addis Ababa	39.36(23.42)	64.42(22.79)	54.21(30.59)	48.85(36.31)	38.61(30.45)	87.95(19.59)
	*P* value	0.231	0.49	0.43	0.49	0.03*	0.79
Marital status	Currently on marriage	39.41 ± (24.76)	62.16(±22.51)	50.45(±32.81)	46.85(±36.04)	41.67(±32.55)	85.81(±20.21)
	Currently not on marriage	42.68(±23.95)	68.69(±22.34)	61.11(±26.3)	53.79(±33.94)	42.93(±31.77)	90.91(± 16.06)
	*P* value	0.431	0.088	0.037*	0.244	0.817	0.104
Educational status	Never go to school	36.55(±23.52	61.53(±23.51)	52.17(±29.2)	43.40(±34.92)	35.42(±30.6)	86.46(19.84)
	Primary	48.26(±22.92)	74.44(±15.44)	63.54(±31.)	65.28(±31.82)	56.25(±33.27)	93.06(±13.83)
	Secondary	54.55(±19.49)	70.91(±21.35)	62.88(±29.7)	60.61(±30.07)	54.55(30.81)	93.94(±11.24)
	12+	51.85(±31.12)	73.33(±22.61)	60.19(±31.39)	68.52(±33.79)	62.96(±23.24)	87.04(±20.03)
	*P* value	0.013*	0.036*	0.296	0.008*	0.002*	0.310
Payment type	Free	40.77(±27.55)	60.00(±24.39)	47.52(±33.90)	38.74(±38.90)	38.29(±37.03)	85.14(±18.75)
	paying	41.02(±23.24)	67.12(±21.72)	58.33(±28.56)	54.21(±32.90)	43.69(±30.17)	89.32(±18.36)
	*P* value	0.957	0.100	0.063	0.021*	0.382	0.239
Care givers relation	suppose	43.00(±24.62)	71.73(±20.93)	53.00(±33.15)	59.33(±34.05)	48.00(±30.18)	93.33(±12.73)
	parents	55.56(±27.0)	68.89(±26.04)	60.19(±20.32)	57.41(±36.4)	53.70(±30.93)	94.44(±11.79)
	children	38.98(±23.39)	62.78(±22.01)	54.86(±31.13)	46.18(±34.67)	38.02(±32.3)	86.63(±20.03)
	sibling	41.67(±29.13)	69.33(±27.97)	63.33(±23.64)	58.33(±39.48)	58.33(±29.66)	85.00(±18.34)
	*P* value	0.257	0.289	0.782	0.278	0.104	0.269
Occupation	Farmer	46.86(±26.51)	72.50(±23.71)	56.77(±30.61)	54.17(±43.67)	44.79(±33.73)	90.62 (±20.16)
	Self employed	45.83(±17.63)	68.33(±18.08)	52.50(±29.01)	50.83(±32.21)	47.50(±30.24)	89.17(±18.16)
	House wife	37.16(±23.32)	61.23(±22.43)	54.41(±30.97)	45.98(±34.19)	36.59(±31.22)	86.59(±18.82)
	Government employ	51.39(±30.33)	76.11(±19.38)	57.64(±30.87)	66.67(±29.30)	58.33(±27.98)	93.06(±16.60)
	Others (retired and employed)	43.33(±36.99)	73.33(±34.32)	76.67(±23.13)	66.67(±40.83)	73.33(±34.55)	93.33(±14.91)
	*P* value	0.191	0.082	0.592	0.268	0.024*	0.704
Cancer type	Cervical, vaginal, vulval	40.10(±24.01)	63.57(±23.35)	54.09(±30.33)	47.77(±36.11)	39.44(±31.08)	87.20(±19.76)
	Ovarian	38.49(±19.98)	66.67(±14.76)	53.57(±28.45)	50.79(±20.74)	42.86(±29.61)	92.06(±12.49)
	Endometrial	61.90(±34.65)	87.62(±19.02)	83.33(±25.00)	85.71(±37.80)	85.71(±26.23)	92.86(±8.91)
	*P* value	0.062	0.022*	0.043*	0.020*	0.001*	0.433
Age	< 40 years	35.18(±24.35)	64.44(±22.75)	46.30(±27.30)	49.08(±31.5)	36.11(±29.29)	87.96(±155.97)
	40-49 years	42.82(±24.73)	68.15(±20.48)	52.08(±30.37)	50.00(±35.41)	43.52(±30.93)	86.11(±18.04)
	50-59 years	39.68(±23.91)	63.97 (±22.48)	55.16 (±28.21)	49.21(±36.97)	36.91(±32.41)	89.68 (±18.01)
	60-69 years	43.33(±23.81)	61.82 (±23.45)	59.00 (±32.80)	53.33(±36.00)	45.33(±32.82)	90.00 (±19.84)
	*P*-value	0.89	0.52	0.079	0.98	0.72	0.73
Parity	0	59.701(±17.01)	85.56(±12.94)	62.50(±13.69)	63.89(±22.15)	63(22.15)	97.22(±6.81)
	1-4	45.74(±25.65)	67.80(±24.01)	61.17(±29.09)	57.44(±33.66)	51.42(±34.01)	87.58(±17.88)
	5-9	36.78(±23.11)	61.33(±21.40)	48.69(±30.05)	43.81(±36.14)	35.24(±29.29)	86.91(20.24)
	≥10	36.79(±23.11)	61.33(±21.40)	48.69(±30.05)	43.81(±36.14)	35.24(±29.29)	86.90(±20.24)
	*P* -value	0.052	0.052	0.063	0.16	0.016	0.46

t test (for two groups comparison) and one way ANOVA (for three and above group comparison) were employed,* significant at *p*-value less than 0.05

**Table 4:** EORTC QLQ-C30 symptom scores by socio-demographic characteristics of gynaecological cancer patients at TASH, Addis Ababa, Ethiopia, 2014 (n = 140)

Variables		Fatigue	Nausea & vomiting	Pain	Dyspnea	Insomnia	Diarrhea	Financial difficulties	constipation	Appetite loss
Address	Addis Ababa	47.86 ± 29.96	7.69 ± 15.69	49.57 ± 30.71	9.40 ± 18.65	25.64 ± 31.03	1.71 ± 7.45	55.56 ± 38.49	38.46 ± 39.40	37.61 ± 39.86
	Out of Addis Ababa	56.33 ± 27.77	8.42 ± 18.80	57.26 ± 29.10	5.61 ± 17.68	40.26 ± 33.77	0.99 ± 7.39	68.32 ± 29.20	46.53 ± 38.90	46.54 ± 35.30
	*P* value	0.116	0.831	0.170	0.265	0.020*	0.607	0.036*	0.275	0.198
Marital status	Currently on marriage	57.81 ± 29.99	9.46 ± 18.93	57.43 ± 30.24	7.66 ± 21.04	40.99 ± 35.99	2.25 ± 10.07	61.26 ± 34.02	45.95 ± 39.29	50.45 ± 38.72
	Currently not on marriage	49.66 ± 26.38	6.82 ± 16.79	52.53 ± 28.98	5.55 ± 13.81	30.81 ± 29.99	0.00	68.69 ± 30.32	42.43 ± 39.03	36.87 ± 33.14
	*P* value	0.092	0.386	0.330	0.492	0.073	0.071	0.177	0.596	0.028*
Educational status	Never go to school	60.53 ± 27.59	7.99 ± 18.57	62.50 ± 29.42	6.94 ± 18.04	43.40 ± 33.90	0.35 ± 3.40	70.49 ± 30.15	53.47 ± 37.61	48.96 ± 36.05
	Primary	41.20 ± 25.48	9.02 ± 17.71	43.06 ± 24.04	0.00 ± 0.00	20.83 ± 21.56	2.78 ± 61	55.55 ± 30.56	29.17 ± 37.19	31.94 ± 31.82
	Secondary	39.39 ± 25.03	9.09 ± 15.57	33.33 ± 19.72	24.24 ± 30.15	21.21 ± 34.23	6.06 ± 13.48	45.45 ± 42.88	15.15 ± 27.34	39.39 ± 38.93
	12+	35.80 ± 28.21	7.41 ± 16.90	35.19 ± 25.61	0.00 ± 0.00	18.52 ± 33.79	0.00 ± 0.00	51.85 ± 33.79	22.22 ± 37.27	29.63 ± 42.31
	*P* value	0.001*	0.991	<0.001*	0.001*	0.002*	0.059	0.015*	<0.001*	0.116
Payment type	Free	59.46 ± 31.67	4.06 ± 9.94	62.61 ± 32.25	3.60 ± 10.49	38.74 ± 35.59	0.90 ± 5.48	73.87 ± 34.37	57.66 ± 41.31	58.60 ± 36.35
	paying	51.99 ± 27.23	9.71 ± 19.87	52.43 ± 28.33	7.77 ± 19.91	35.28 ± 32.95	1.29 ± 7.98	61.49 ± 31.23	39.48 ± 37.27	38.83 ± 35.58
	*P* value	0.173	0.100	0.073	0.228	0.592	0.782	0.046*	0.015*	0.005*

t test (for two groups comparison) and one way ANOVA (for three and above group comparison) were employed,,* significant at *p*-value less than 0.05

Patients coming from outside Addis Ababa had significantly lower score for social function compared to those who were from Addis Ababa (*p* = 0.03)(Table 3). Insomnia (*p* = 0.02), and financial difficulties (*p* = 0.036) were significantly higher in those patients who were coming from outside Addis Ababa (Table 4). Patients who never went to school had significantly lower score in GHS (*p* = 0.013), physical function (*p* = 0.036), role function (*p* = 0.008) and social function (*p* = 0.002) (Table 4). Those patients who never went to school had significantly higher score for symptom scales such as fatigue (*p* = 0.001), pain (*p* < 0.001), dyspnea (*p* = 0.001), insomnia (*p* = 0.002), financial difficulties (*p* = 0.015), and constipation (*p* < 0.001) (Table 4).

Marital status was significantly associated with emotional function and appetite loss. This study found that those married patients had significantly higher score on emotional function than their unmarried counter parts (*p* = 0.037). Appetite loss score was significantly higher among those married patients (*p* = 0.028) than their unmarried counter parts. The score for role function among patients who were paying was significantly higher as compared to patients who were free of charge (*p* = 0.021). Financial difficulties (*p* = 0.046), constipation (*p* = 0.015) and appetite loss (*p* = 0.005) were also scored low among paying group (Table 3 & 4). Parity was significantly associated with social function (*p* = 0.016), fatigue (*p* = 0.015), pain (*p* = 0.024), financial difficulties (*p* = 0.003), appetite loss (*p* = 0.003). This study found that EORTC-Q30 functional scale domains varied significantly depending on the gynecologic cancer type. Endometrial cancer was associated with a better score in GHS; in all functional scale (*P* value ≤0.05) except cognitive function (Table 3) and in all symptom scales domains (*p* ≤ 0.05) except constipation, diarrhea and insomnia (Table 5). Cervical cancer patients had significantly worse score in fatigue and pain (*p* ≤ 0.05). As shown on Table 5, having ovarian cancer was associated with a higher score in dyspnea and nausea/vomiting than other types of cancers (p ≤ 0.05). Endometrial cancer has a better score in fatigue, pain and financial difficulties as compared to the other cancer types (Pp ≤ 0.05) (Table 5).

**Table 5:** EORTC QLQ-C30 symptom scores by clinical characteristics of gynaecological cancer patients at TASH, Addis Ababa, Ethiopia, 2014(n = 140)

Variables		Fatigue	Nausea & vomiting	Pain	Dyspnea	Insomnia	Diarrhea	Financial difficulties	constipation	Appetite loss
Parity	0	25.92(±15.18)	5.56(±13.61)	33.33(±14.91)	16.67(±27.89)	16.67(27.89)	0.00(±0.00)	27.78(±32.78)	11.11(±27.22)	38.89(±38.97)
	1-4	48.69(±29.36)	6.74(15.01)	47.87(±29.20)	5.67(±17.47)	31.91(36.08)	1.41(±6.80)	58.87(±33.48)	37.58(±39.69)	32.62(36.44)
	5-9	59.68(±26.77)	10.71(±21.43)	61.43(28.31)	7.14(±18.73)	39.99(±31.90)	1.43(±8.86)	72.38(±28.36)	50.48(±36.66)	55.24(±34.46)
	≥10	54.90(±30.30)	2.94(±6.55)	56.86(±33.88)	3.92(±11.07)	39.21(±33.82)	0.00(±0.00)	62.74(±35.12)	49.01(±44.28)	31.37(±34.33)
	*P* value	0.015	0.35	0.024	0.49	0.28	0.87	0.003	0.05	0.003
Stage	Stage 1	32.41(±26.58)	9.72 ± 28.83)	33.34(±22.47)	2.78(±9.62)	25.00 ± 28.87	0.00 ± 0.00	55.55 ± 38.49	22.22 ± 41.03	22.22 ± 38.48
	Stage 2	49.43(±24.33)	3.40(±8.32)	50.68(±26.78)	4.08(±12.97)	27.89 ± 29.93	0.68 ± 4.76	67.35 ± 30.80	33.33 ± 34.70	36.06 ± 31.80
	Stage 3 and 4	66.07 ± 28.70	8.93 ± 19.846	70.54 ± 27.34	7.14 ± 19.81	49.41 ± 35.39	1.78 ± 9.89	70.24 ± 28.20	63.69 ± 36.11	51.19 ± 37.05
	*P*-value	<0.001*	0.219	<0.001*	0.536	0.002*	0.649	0.320	<0.001*	0.013*
Cancer type	Cervical, vaginal vulvar	55.95 ± 29.11	5.51 ± 14.91	58.78 ± 29.17	4.46 ± 15.17	37.80 ± 34.51	0.89 ± 7.02	67.56 ± 30.50	46.73 ± 39.13	41.96 ± 36.00
	Ovarian	53.97 ± 20.87	24.60 ± 25.61	46.83 ± 26.15	20.64 ± 26.83	33.33 ± 27.89	3.17 ± 10.03	58.73 ± 36.37	39.68 ± 40.30	61.91 ± 33.81
	Endometrial	22.22 ± 22.22	2.38 ± 6.30	21.43 ± 23.00	0.00 ± 0.00	19.05 ± 32.53	0.00 ± 00.00	38.10 ± 40.50	19.05 ± 26.23	23.81 ± 41.79
	*P* value	0.009*	0.000*	0.002*	0.000*	0.330	0.394	0.042*	0.161	0.023*

Independent t test (for two groups comparison) and one way ANOVA (for three and above group comparison) were employed,,* significant at *p*-value less than 0.05

## Discussion

Social function was the most affected while cognitive function was the least affected domain among the functional scale in gynaecological cancer patients in Ethiopia. Among symptom scale, financial difficulty was the worst affected QOL dimension. Studies conducted in other developing countries, Tanzania and Indonesia showed that financial difficulty is one of the most affected domain among the symptom scale [8, 9]. We found that pain and fatigue were the second and the third highly scored domains whereas a Turkish and Malaysian studies showed that either of the two are the most affected domains [10, 11, 12]. This may be due to the fact of inaccessibility of the oncology service and lack of health insurance in the Ethiopian settings. This makes patients to travel over a long distance to get oncologic service which could be the cause of fatigue and pain. The mean score for GHS in this study was 40.95 which is a little lower than the Tanzanian report (50.5) performed amongst all types of cancer [8]. This difference could be attributed to the difference in socio-demographic characteristics, cancer type and health services access between Ethiopia and Tanzania.

This study found that among the socio demographic characteristics, marital status was significantly associated with emotional function and appetite loss. However, elsewhere marital status has shown to have inconsistent association with quality of life. In one Iranian study [13] marital status was found not correlated in any of the scores where as a study done in Turkey [10, 14] showed married participants to have a low score in emotional function in contrary to our finding.

Patients from outside Addis Ababa have a lower score of social function and a higher score of insomnia and financial difficulties. Traveling from home to Addis Ababa, where the service is available creates additional expense for traveling; the need for somebody to accompany them and the need to stay in a new environment during the treatment period. This could affect their social function, insomnia and financial difficulties. Additional evidence that showed financial difficulties can compromise other quality of life domain is that those patients who were being treated free of charge because they are poor have low score of role function and high score in financial difficulty, high score in constipation and high score in appetite loss.

Educational status has also shown a significant correlation with health related quality of life components. Those patients who had never gone to school scored lower in GHS, physical function, and social function and had higher score in fatigue, pain, dyspnea, financial difficulties and constipations. A study done in Turkey showed education status was associated with physical function and pain in the same way as ours but in contrary to ours with fatigue [10]. This can be justified by the fact that a lower level of education is associated with poor health seeking behavior. This study also found that those patients who are house wife had a lower score in social function. There was a similar finding from Sudan which showed higher QOL of scores for patients when they are employed in medium skill/high skill occupation [15].

The EORTC-Q30 scores varied based on cancer type within the broad category of Gynaecologic cancer. Endometrial cancer patients scored better in most of the functional and symptom scale domains. This can be explained by the early presentation of the endometrial cancers as compared to the others. The Turkish study has also shown endometrial cancer to be favored in role function, social function, fatigue and financial difficulties [10]. Stage of the disease and the intended type of treatment were associated with quality of life of cervical cancer patients. Advanced stage of diseases and radiation treatment found associated with the worst side [7]. A study done in Texas has described cervical cancer survivors treated with radiotherapy to report more QOL impairments than survivors treated with other approaches [16].

### Limitation of the study

This study had the following major limitations. This study did not employed multivariable regression analysis to control the confounders because we have many scores used as indicator of quality of life. The study was conducted using Amharic version of EORTC QLQ-C30 despite the fact that all study participants were not Amharic native speakers. EORTC QLQ-C30 questionnaire was also not tested for its responsiveness and test-retest reliability.

## Conclusion

GHS, social function, financial difficulties, pain and fatigue were the most affected however cognitive function and diarrhea were less affected components of HRQOL of gynaecological cancer patients. Place of residence, educational status, marital status, payment type, cancer type and stage of the disease were some of the different socio demographic and disease related factors were associated with health related quality of life.

## Abbreviations

ANOVA: Analysis of Variance
EORTC QLQ- C30: European Organization for Research and Treatment of Cancer Quality of life questionnaire core-30
GHS: Global Health Function
HRQOL: Health Related Quality of Life
QOL: Quality of life
TASH: Tikur Anebesa Specialized Hospital
WHO: World Health Organization
